# A118 DE NOVO CROHN’S DISEASE FOLLOWING ORTHOTOPIC LIVER TRANSPLANTATION IN A PATIENT WITH PRIMARY SCLEROSING CHOLANGITIS: A CASE REPORT

**DOI:** 10.1093/jcag/gwae059.118

**Published:** 2025-02-10

**Authors:** A Bello, N Samad

**Affiliations:** University of Saskatchewan, Saskatoon, SK, Canada; University of Saskatchewan, Saskatoon, SK, Canada

## Abstract

**Background:**

Primary sclerosing cholangitis (PSC) is strongly associated with inflammatory bowel disease (IBD), most commonly ulcerative colitis, with up to 80% of PSC patients developing IBD during their lifetime. However, the development of de novo Crohn’s disease following orthotopic liver transplantation (OLT) is rare, complicating both immunosuppressive management and long-term patient outcomes.

**Aims:**

The aim of this study is to report a rare case of de novo Crohn’s disease post-OLT in a PSC patient, exploring their presentation, the role of tacrolimus in its development and highlighting successful management with vedolizumab.

**Methods:**

We conducted a retrospective case analysis of a 44-year-old female patient who underwent OLT in 2021 for PSC-related decompensated cirrhosis. Prior to the transplant, the patient had no history of IBD, and a screening colonoscopy revealed normal colonic mucosa, although histological analysis indicated proctitis. Approximately two years post-OLT, while on the immunosuppresive agent tacrolimus, the patient developed new gastrointestinal symptoms, including abdominal pain, diarrhea, tenesmus, and nocturnal bowel movements. Diagnostic evaluation included endoscopic assessment and histopathological analysis of biopsy samples.

**Results:**

Endoscopic examination revealed inflammation characterized by ileitis and left-sided colitis. Histopathology confirmed a diagnosis of de novo Crohn’s disease. The patient’s clinical course was complicated by the need to balance her immunosuppressive therapy with treatment for Crohn’s disease. Vedolizumab, a gut-selective biologic agent targeting the α4β7 integrin, was initiated alongside her existing immunosuppressive regimen. Over the course of treatment, the patient demonstrated significant clinical improvement, with a marked reduction in abdominal pain and diarrhea, as well as an overall improvement in quality of life.

**Conclusions:**

The development of de novo Crohn’s disease in patients with PSC following OLT, though rare, presents a complex clinical challenge. Immunosuppressive agents, particularly tacrolimus, may play a contributory role in the pathogenesis of de novo IBD, though the precise mechanisms remain unclear. Early diagnosis and intervention with targeted therapies, such as vedolizumab, are critical in managing IBD in this population, as they allow for effective control of gastrointestinal inflammation while maintaining necessary immunosuppression. This case underscores the importance of vigilant surveillance in post-OLT patients to ensure timely intervention. Further research is warranted to elucidate the underlying risk factors and optimize therapeutic strategies for de novo IBD in the post-transplant setting, particularly in PSC patients who may be at increased risk.

**Funding Agencies:**

None

